# Impact of recipient and donor pretransplantation body mass index on early postosperative complications after lung transplantation

**DOI:** 10.1186/s12890-024-02977-z

**Published:** 2024-04-03

**Authors:** E. Atchade, C. De Tymowski, E. Lepitre, N. Zappella, A. Snauwaert, S. Jean-Baptiste, A. Tran-Dinh, B. Lortat-Jacob, J. Messika, H. Mal, P. Mordant, Y. Castier, S. Tanaka, P. Montravers

**Affiliations:** 1 DMU PARABOL, APHP, CHU Bichat-Claude Bernard, Département d’anesthésie Reanimation, 46 Rue Henri Huchard, 75018 Paris, France; 2 UMR 1149, INSERM, Immunorecepteur Et Immunopathologie Rénale, CHU Bichat-Claude Bernard, 46 Rue Henri Huchard, 75018 Paris, France; 3 INSERM U1148, LVTS, CHU Bichat-Claude Bernard, 46 Rue Henri Huchard, 75018 Paris, France; 4 UMR 1188, Université de La Réunion, INSERM, Diabète Athérothrombose Réunion Océan Indien (DéTROI), Saint-Denis de La Réunion, France; 5 Service de Pneumologie B Et Transplantation Pulmonaire, APHP, CHU Bichat-Claude Bernard, 46 Rue Henri Huchard, 75018 Paris, France; 6 Université de Paris Cité, UFR Diderot, Paris, France; 7 Service de Chirurgie Thoracique Et Vasculaire, APHP, CHU Bichat-Claude Bernard, 46 Rue Henri Huchard, 75018 Paris, France; 8 UMR 1152ANR-10LABX17Physiopathologie Et Epidémiologie Des Maladies Respiratoires, INSERM, Paris, France

**Keywords:** Lung transplantation, Body mass index, Outcome, Intensive care unit, Primary graft dysfunction, Donor acceptability criteria

## Abstract

**Background:**

Prior studies have assessed the impact of the pretransplantation recipient body mass index (BMI) on patient outcomes after lung transplantation (LT), but they have not specifically addressed early postoperative complications. Moreover, the impact of donor BMI on these complications has not been evaluated. The first aim of this study was to assess complications during hospitalization in the ICU after LT according to donor and recipient pretransplantation BMI.

**Methods:**

All the recipients who underwent LT at Bichat Claude Bernard Hospital, Paris, between January 2016 and August 2022 were included in this observational retrospective monocentric study. Postoperative complications were analyzed according to recipient and donor BMIs. Univariate and multivariate analyses were also performed. The 90-day and one-year survival rates were studied. *P* < 0.05 was considered to indicate statistical significance. The Paris-North Hospitals Institutional Review Board approved the study.

**Results:**

A total of 304 recipients were analyzed. Being underweight was observed in 41 (13%) recipients, a normal weight in 130 (43%) recipients, and being overweight/obese in 133 (44%) recipients. ECMO support during surgery was significantly more common in the overweight/obese group (*p* = 0.021), as were respiratory complications (primary graft dysfunction (PGD) (*p* = 0.006), grade 3 PDG (*p* = 0.018), neuroblocking agent administration (*p* = 0.008), prone positioning (*p* = 0.007)), and KDIGO 3 acute kidney injury (*p* = 0.036). However, pretransplantation overweight/obese status was not an independent risk factor for 90-day mortality. An overweight or obese donor was associated with a decreased PaO2/FiO2 ratio before organ donation (*p* < 0.001), without affecting morbidity or mortality after LT.

**Conclusion:**

Pretransplantation overweight/obesity in recipients is strongly associated with respiratory and renal complications during hospitalization in the ICU after LT.

**Supplementary Information:**

The online version contains supplementary material available at 10.1186/s12890-024-02977-z.

## Introduction

Body mass index (BMI) is the reference tool that defines underweight, normal weight, overweight and obese individuals according to the World Health Organization [1]. In lung transplantation (LT), the 2021 report of the International Society for Heart and Lung Transplantation (ISHLT) described that an abnormal pretransplantation BMI was present in 55% of the LT recipients in the 2010–2018 period [2].

The impact of abnormal pretransplantation BMI on outcomes after LT has already been studied. Most of the prior studies described an increased mortality rate in recipients with an abnormal pretransplantation BMI. The last ISHLT report described a significant association between a pretransplantation BMI > 30 kg/m2 and one-year mortality [2], as did an analysis of the UNOS database [3]. These findings confirmed prior monocentric studies [4–7]. Another large cohort assessing 11,411 LT recipients showed increased 30-day mortality in recipients with an abnormal BMI (underweight or obesity) in comparison with recipients with a normal BMI [8].

However, the threshold above which an abnormal BMI affects mortality has been reported to vary among different studies. This can probably be explained by the limits of the BMI, which does not take into account body composition, sex, age or ethnicity, to diagnose underweight of overweight / obesity. Singer et al. observed a significant effect of increased pretransplantation BMI on mortality only in patients with class II (BMI > 35 kg/m2) or III (BMI > 40 kg/m2) obesity [9]. The underlying mechanisms of this overmortality are not clear, but early postoperative complications are suspected. In overweight or class I obesity patients, increased morbidity (increased duration of surgical procedure, duration of cold ischemia, postoperative atelectasis and impaired cachexia) was observed without a significantly increased mortality rate [9]. Some studies have even shown a reduced postoperative mortality rate in overweight patients [10, 11].

As a consequence, the ISHLT guidelines recommend considering class I obesity (BMI 30–34.9 kg/m^2^) as a relative contraindication to LT and class II and III obesity (BMI ≥ 35 kg/m^2^) as absolute contraindications [12].

Interestingly, little is known about the effect of donor BMI on early postoperative complications and mortality rates after LT. Ideal acceptability criteria for human lung donors do not include BMI criteria [13, 14], and to our knowledge, no prior study has specifically assessed the impact of a donor’s overweight status and obesity on early postoperative complications after LT.

The main objective of this study was to assess postoperative complications during hospitalization in the ICU after LT according to the donor’s and the recipient’s pretransplantation BMI. The secondary aim was to evaluate the 90-day and one-year mortality rates of LT recipients stratified by their pretransplantation BMI.

## Materials and methods

### Study population

This observational, monocentric study was a retrospective analysis of a prospectively implemented database. All the recipients who underwent LT at Bichat Claude Bernard Hospital, APHP, Paris, between January 2016 and August 2022 were included in the study. The study was reviewed and approved by the Paris-North-Hospitals Institutional Review Board (Paris Diderot University, AP-HP, IRB No. 00006477) who waived the need for an informed consent because of the observational nature of the study, according to French law.

### Data collection

The following data were recorded: characteristics of the recipients (demographic data, indication for LT, comorbidities, pretransplantation BMI, use of ECMO as a bridge to LT), and donor information (age, sex, BMI, duration of mechanical ventilation (MV), PaO2/FiO2 ratio before organ donation, transfusion, and tobacco use). Patient data concerning the characteristics of the LT procedure (the nature of the surgical procedure and duration, peridural anesthesia, hemodynamic status and transfusion during surgery) were also collected. Postoperative complications during hospitalization in the ICU were recorded, including severity scores and lactateaemia at ICU admission; respiratory complications (primary graft dysfunction (PGD) and grade; duration of MV; neuroblocking agent (NBA) administration; prone positioning; tracheostomy for ventilation weaning; hemodynamic and renal complications (ECMO and catecholamine support after surgery and duration; AKI); infectious, surgical and airway complications; and acute rejection. The short-term outcomes of the recipients (duration of hospitalization in the ICU, death on Day 90 and at one year) were also recorded.

### Definitions

Comparisons of postoperative complications were performed between 3 groups of recipients (underweight [BMI < 18.5 kg/m^2^], normal BMI [18.5–24.9] kg/m^2^] and overweight/obese [BMI ≥ 25 kg/m^2^]) [1] and 4 groups of donors (underweight [BMI < 18.5 kg/m^2^], normal weight [BMI [18.5–24.9] kg/m^2^], overweight [BMI [25–29.9] kg/m^2^]), and obese [BMI ≥ 30 kg/m^2^]). Primary graft dysfunction (PGD) was defined and graded according to the ISHLT definition [15]. AKI was defined according to the Kidney Disease: Improving Global Outcome (KDIGO) definition [16]. Septic shock was defined according to the Sepsis-3 definition [17]. Acute rejection was defined according to the ISHLT nomenclature [18].

### Perioperative care of the recipients

Inscription on the waiting list was provided by a multidisciplinary team (pulmonologist, thoracic surgeon, anesthesiologist, intensivist) in accordance with the ISHLT guidelines [12].

The perioperative management of the recipients, during and after surgery, was standardized according to our local protocol [19, 20]. ECMO support strategy is protocolized in our centre. Before surgery, ECMO is implemented as a bridge to LT if severe hypoxemia persists despite high-flow oxygen therapy. Veno-venous ECMO is favoured in the absence of severe arterial pulmonary hypertension (mean pulmonary arterial pressure (PAP) > 50 mmHg). During the intraoperative period, if applicable, veno-venous ECMO as a bridge to LT is most often converted into a venoarterial device. Venoarterial ECMO support is also required in cases of severe pulmonary hypertension, preexisting or perioperative right-sided cardiac dysfunction, or when the patient does not tolerate single-lung ventilation (mean PAP > 50 mmHg, SaO2 < 85%, SvO2 < 60%, cardiac output < 1.5 L/min/m2, hypercapnia).

### Statistical analysis

Qualitative variables are expressed as absolute numbers and percentages, and quantitative data are expressed as medians and interquartile ranges. To assess early postoperative complications, univariate analysis was performed using the chi-square or Mann–Whitney U test, as appropriate. The 90-day and one-year survival rates stratified by donor and recipient BMIs were studied using Kaplan‒Meier curves and log rank tests. To study the independent risk factors for 90-day mortality, a multivariate analysis was performed. Variables with a p value < 0.2 in univariate analysis were entered into a backward stepwise logistic regression model. When several related variables were associated with 90-day mortality according to univariate analysis, the most clinically relevant variables were included in the multivariate model. Analysis of postoperative complications in the subgroups of COPD and pulmonary fibrosis were also performed. A *p* < 0.05 was defined as significant. Statistical analysis was performed using R. (R Foundation for Statistical computing, Vienna, Austria, http://www.R-project.org/).

## Results

### General characteristics of the study population

**Fig.1:**
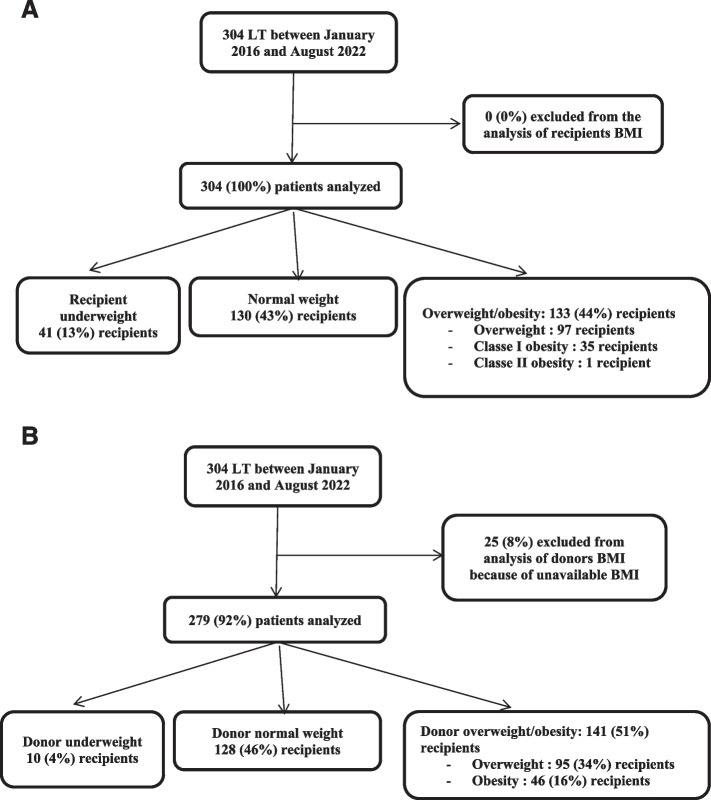
Flow chart of the study. **A** Analysis of recipient pretransplantation BMI. **B** Analysis of donor BMI

**Fig.2:**
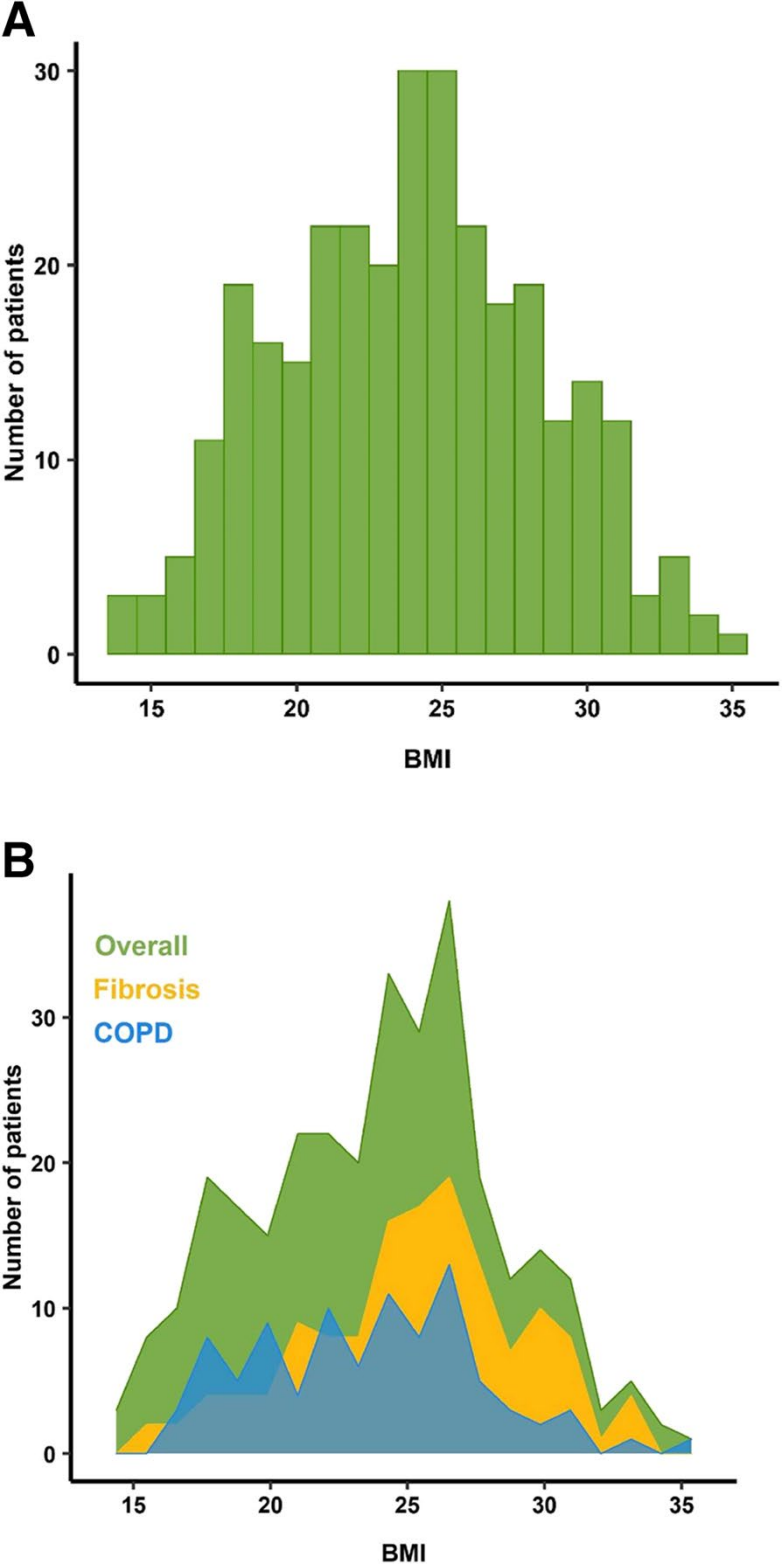
**A** Distribution of pretransplantation BMI in the recipient population. **B** Distribution of recipient pretransplantation BMI according to the diagnosis of diagnosis leading to LT

Overall, 304 patients who underwent LT in Bichat Claude Bernard hospital between January 2016 and August 2022 were included in the analysis of postoperative complications depending on recipients pretransplantation BMI. However, 25 recipients were excluded of analysis of donor’s BMI, as this data was not available. Median BMI in the all cohort was 24[21-27] Kg/m^2^ for the recipients, and 24[21-27] Kg/m^2^ for donors. Median weight of recipients was 70[58–80] Kg. 41 (13%) recipients were underweighted, 130 (43%) recipients had a normal pretransplantation BMI, and 133 (44%) recipients were overweighted or obese (36 (12%) obese recipients, including 35 class I obesity (BMI 30–34.9 kg/m^2^)). The flow chart of the study is presented in Fig. 1A and B. The distribution of recipients pretransplantation BMI and their distribution depending on diagnosis leading to LT are presented in Fig. 2A and B.

### General characteristics of the grafts depending on recipient’s pretransplantation BMI

The general characteristics of the grafts depending on recipient’s and donor’s BMI are presented in Table 1. The median PaO2/FiO2 ratio before organ donation was significantly lower in overweighted or obese donors (*p* < 0.001).

**Table 1:** Characteristics of the grafts depending on the recipient’s and donor’s BMI, univariate analysis

	**Recipient BMI**				**Donor BMI**				
	**Underweight** ***N* = 41 (13%)**	**Normal weight** ***N* = 130 (43%)**	**Overweight/obesity** ***N* = 133 (44%)**	***p***	**Underweight**, *N* = 10 (3.6%)	**Normal weight**, *N* = 128 (46%)	**Overweight,** *N* = 95 (34%)	**Obesity**, *N* = 46 (16%)	***p***
Male gender, n (%)	11 (27)	72 (55)	75 (56)	0.002	6 (60)	61 (48)	56 (59)	25 (54)	0.38
Duration of MV, donor, med [IQR]	2 [1, 4]	2 [1, 3]	2 [1, 3]	0.75	2 [1–3]	2 [1–3]	2 [2–4]	2 [1–3]	0.21
PaO2/FiO2, donor, med [IQR]	402 [350–470]	380 [326–450]	390 [334- 464]	0.38	482 [401–499]	425 [351–481]	379 [326–446]	345 [302–380]	< 0.001
Age, donor, med [IQR]	51 [44–57]	52 [35–62]	54 [42–63]	0.56	45 [27–50]	49 [35–60]	55 [47–65]	55 [46–59]	0.002
Tobbaco use, donor, n (%)	18 (45)	35 (28)	65 (50)	0.001	2 (20)	52 (42)	33 (35)	20 (43)	0.43
Transfusion, donor, n (%)	11 (27)	37 (29)	33 (26)	0.84	1 (10)	38 (30)	24 (26)	8 (18)	0.31

Quantitative variables were compared using Mann-Withney U test; qualitative datas using Chi-2 tests

*BMI* body mass index, *IQR* interquartile range, *MV* mechanical ventilation

### Characteristics of the recipients before and during surgical procedure depending on their recipients and donors BMI

The characteristics of the recipients before and during the surgical procedure depending on recipient’s pretransplantation BMI and donor’s BMI are presented respectively in Tables 2 and 3. The recipient’s comorbidities (diabetes mellitus, high blood pressure, hypercholesterolemia, chronic ischemic heart disease) were more frequent in the overweighted/obese recipients.The need for extracorporeal membrane oxygenation (ECMO) support during surgery was significantly more frequent in overweighted/obese recipients (80% versus 66% in underweighted or normal weight recipients, p = 0.021).

**Table 2:** Characteristics of the recipients and intra-operative period depending on recipient’s pretransplantation BMI, univariate analysis

	All cohort, *N* = 304 (100)	Recipient underweight, N = 41 (13%)	Recipient normal weight, *N* = 130 (43%)	Recipient overweight/obesity, *N* = 33 (44%)	p
Characteristics of the recipients					
Age, recipient, years,	57[50–62]	51 [30–58]	57 [51–62]	57 [52–62]	< 0.001
Male gender, n (%)	197 (65)	17 (41)	82 (63)	98 (74)	< 0.001
Height, cm, med [IQR]	170 [163–176]	162 [158–170]	170 [163–176]	172 [166–178]	< 0.001
Weight, Kg, med [IQR]	70 [58–80]	45 [43–50]	63 [56–70]	80 [75–89]	< 0.001
BMI, Kg/m^2^, med [IQR]	24 [21–27]	17 [16–18]	22 [20.9–24]	27.7 [9, 26–29]	< 0.001
Smoking history, recipient, n (%)	208 (68)	22 (54)	89 (68)	97 (73)	0.068
Underlying disease					
Pulmonary fibrosis, n (%)	136 (45)	9 (22)	50 (38)	77 (58)	< 0.001
COPD, n (%)	92 (30)	11 (27)	45 (35)	36 (27)	0.36
Comorbidities					
Diabetes mellitus, n (%)	33 (11)	7 (17)	9 (7)	17 (13)	0.097
High blood pressure, n (%)	77 (25)	7 (17)	24 (18)	46 (35)	0.005
Hypercholesterolemia, n (%)	65 (21)	5 (12)	22 (17)	38 (29)	0.021
Chronic ischaemic heart disease, n (%)	27 (9)	1 (2)	5 (4)	21 (16)	0.001
Peripheral arterial disease, n (%)	13 (4)	1 (3)	4 (3)	8 (6)	0.53
Preoperative assessment					
Pulmonary hypertension, n (%)	149 (50)	16 (42)	65 (50)	68 (52)	0.57
Dilatation of the right ventricle, n (%)	82 (27)	8 (20)	37 (29)	37 (28)	0.50
Left ventricular ejection fraction, %, med [IQR]	63 [58–68]	62 [58–66]	64 [60–69]	62 [58–67]	0.47
Serum creatinin, µmol/L, med [IQR]	68 [56–80]	53 [49–62]	68 [55–80]	72 [62–87]	< 0.001
Clinical characteristics before surgery					
High flow oxygenotherapy before surgery, n (%)	50 (16)	7 (17)	18 (14)	25 (19)	0.55
ECMO as bridge to LT, n (%)	23 (8)	5 (12)	5 (4)	13 (10)	0.075
High emergency LT, n (%)	59 (19)	11 (27)	22 (17)	26 (20)	0.38
Characteristics of intra-operative period					
Bilateral LT, n (%)	210 (69)	30 (73)	94 (72)	86 (65)	0.34
Retransplantation, n (%)	9 (3)	3 (7)	5 (4)	1 (1)	0.048
Duration of surgical procedure, med [IQR]	420 [315–490]	420 [315–500]	420 [380–480]	420 [350–505]	0.78
Peridural anesthesia, n (%)	197 (66)	24 (60)	88 (69)	85 (64)	0.55
ECMO support during surgery, n (%)	220 (72)	27 (66)	86 (66)	107 (80)	0.021
ECMO weaned in operating room, n (%)	130 (59)	18 (67)	54 (63)	58 (54)	0.34
Cathecholamine support during surgery, n (%)	295 (97)	41 (100)	123 (95)	131 (98)	0.12
Transfusion during surgery, n (%)					
RBC transfusion	204 (68)	32 (78)	89 (69)	83 (63)	0.17
0 RBC unit	98 (32)	9 (22)	42 (33)	47 (36)	0.30
1–4 RBC units	148 (49)	20 (49)	64 (50)	64 (48)	
≥ 5 RBC units	56 (19)	12 (29)	23 (18)	21 (16)	
FFP transfusion	188 (62)	30 (73)	74 (57)	84 (63)	0.16
Platelet transfusion	64 (21)	11 (27)	31 (24)	22 (17)	0.22
Vascular filling ≥ 2500 mL during surgery, n (%)	260 (86)	35 (85)	114 (88)	111 (85)	0.78

*BMI* body mass index, *IQR* interquartile range, *ECMO* extra-corporeal membrane oxygenation, *LT* lung transplantation, *MV* mechanichal ventilation, *RBC* red blood cell, *FFP* fresh frozen plasma

**Table 3:** Characteristics of the recipients and intra-operative period depending on donors BMI, univariate analysis

	Overall, *N* = 279 (100%)	Donor underweight, *N* = 10 (3.6%)	Donor normal weight, *N* = 128 (46%)	Donor overweight, *N* = 95 (34%)	Donor obesity, *N* = 46 (16%)	*p*-value
Before surgery						
Age, recipient, n (%)	57 [51–62]	56 [45–59]	57 [50–62]	57 [51–62]	58 [53–63]	0.46
Male gender, recipient, n (%)	179 (64)	5 (50)	83 (65)	62 (65)	29 (63)	0.81
Height, cm, med [IQR]	170 [163–176]	166 [159–175]	171 [164–176]	170 [163–177]	170 [164–177]	0.81
Weight, Kg, med IQR]	69 [58- 80]	57 [46–78]	71 [59–82]	67 [57–79]	70 [60–80]	0.27
BMI, Kg/m2, med[IQR]	24 [21–27]	21 [18–24]	24 [21–27]	23.9 [19–27]	24 [22–28]	0.19
Diabetes mellitus, n (%)	30 (11)	3 (30)	15 (12)	10 (11)	2 (4)	0.12
High blood pressure, n (%)	70 (25)	5 (50)	29 (23)	23 (24)	13 (28)	0.27
Dyslipidemia, n (%)	57 (20)	3 (30)	27 (21)	19 (20)	8 (17)	0.79
Ischc cardiopathy	27 (10)	1 (10)	11 (9)	10 (11)	5 (11)	0.91
Underlying disease						
COPD, n (%)	86 (31)	1 (10)	37 (29)	34 (36)	14 (30)	0.37
PF, n (%)	130 (47)	7 (70)	65 (51)	35 (37)	23 (50)	0.071
Other, n (%)	55 (20)	2 (20)	21 (16)	22 (23)	10 (22)	0.59
High flow oxygenotherapy before surgery, n (%)	46 (16)	3 (30)	22 (17)	15 (16)	6 (13)	0.59
MV before surgery, n (%)	4 (1.4)	0 (0)	1 (1)	3 (3.2)	0 (0)	0.47
ECMO support before surgery, n (%)	21 (8)	2 (20)	8 (6)	8 (8)	3 (7)	0.38
Tobacco use, n (%)	190 (68)	5 (50)	84 (66)	70 (74)	31 (67)	0.34
Peripheral arterial disease, n (%)	12 (4)	0 (0)	7 (6)	5 (5)	0 (0)	0.41
Pulmonary arterial hypertension, n (%)	134 (49)	5 (50)	63 (50)	44 (47)	22 (49)	0.97
Dilatation of the right ventricle, n (ù°	77 (28)	3 (30)	32 (25)	32 (34)	10 (22)	0.36
Ejection fraction of the left ventricle, %, med [IQR]	62 [58–68]	60 [58–64]	63 [58–69]	61 [60–65]	63 [55–68]	0.15
Serum creatinine, med [IQR]	69 [56–80]	57 [54–76]	72 [56–84]	65 [53–78]	71 [59–83]	0.083
High emergency LT, n (%)	53 (19)	3 (30)	23 (18)	19 (20)	8 (17)	0.74
Retransplantation	7 (2.5)	1 (10)	4 (3)	0 (0)	2 (4.3)	0.067
Redux	27 (9.9)	2 (22)	16 (13)	4 (4.3)	5 (11)	0.066
During surgery						
Bilateral LT, n (%)	193 (69)	6 (60)	88 (69)	70 (74)	29 (63)	0.52
Duration of cold ischemia, min, first lung, med [IQR]	277 [240–334]	355 [285–420]	280 [240–342]	270 [226–330]	278 [240–340]	0.25
Duration of cold ischemia, min, second lung, med [IQR]	375 [308–427]	428 [332–434]	390 [315–448]	360 [300–408]	365 [300–420]	0.20
Duration of anesthesia, min, med [IQR]	420 [360–500]	390 [360–420]	420 [370–500]	420 [360–500]	410 [353–498]	0.66
Peridural anesthesia, n (%)	185 (67)	4 (40)	86 (68)	62 (67)	33 (73)	0.26
ECMO support during surgery, n '(%)	202 (72)	7 (70)	96 (75)	62 (65)	37 (80)	0.23
Catecholamine support during surgery, n (%)	273 (98)	10 (100)	126 (98)	92 (97)	45 (98)	0.89
RBC transfusion during surgery, n (%)	188 (68)	9 (90)	85 (67)	66 (69)	28 (62)	0.40
0 RBC unit, n (%)	88 (32)	1 (10)	42 (33)	29 (31)	16 (36)	
1–4 RBC units, n (%)	137 (49)	7 (70)	64 (50)	45 (47)	21 (47)	
≥ 5 RBC units, n (%)	52 (19)	2 (20)	21 (17)	21 (22)	8 (18)	
Fresh frozen plasma transfusion, n (%)	175 (63)	6 (60)	79 (62)	61 (64)	29 (63)	0.98
Platelet transfusion n (%)	56 (20)	5 (50)	24 (19)	16 (17)	11 (24)	0.094
Vascular filling > 2500 mL, n (%)	236 (85)	10 (100)	104 (83)	84 (88)	38 (83)	0.37

Quantitative variables were compared using Mann-Withney U test; qualitative datas using Chi-2 tests

*BMI* body mass index, *IQR* interquartile range, *ECMO* extra-corporeal membrane oxygenation, *LT* lung transplantation, *MV* mechanichal ventilation, *RBC* red blood cell, *FFP* fresh frozen plasma

### Early postoperative complications of the recipients depending on recipients and donors BMI

The postoperative complications of the recipients during hospitalization in ICU depending on recipient’s and donor’s BMI are presented respectively in Tables 4 and 5.

**Table 4:** Postoperative complications during hospitalization in the ICU and short-term outcomes of the recipients stratified by preoperative BMI; univariate analysis

	Overall, *N* = 304 (100%)	Recipient underweight *N* = 41 (13%)	Recipient normal weight *N* = 130 (43%)	Recipient overweight/obesity, *N* = 133 (44%)	*p *value
At admission in ICU					
SAPS II score, med [IQR]	44 [39–53]	44 [37–50]	44 [38–52]	44 [39–54]	0.28
SOFA score, med [IQR]	8 [6–10]	8 [6–9]	8 [6–10]	8 [7–10]	0.23
Lactatemia > 3 mmol/L, n (%)	108 (36)	16 (39)	42 (32)	50 (38)	0.59
Lactatemia > 2 mmol/L, n (%)	185 (61)	29 (71)	76 (58)	80 (60)	0.36
Hemodynamic status during hospitalization in ICU					
Duration of catecholamine administration, days, med [IQR]	2 [1–4]	2 [1–3.5]	2 [1–4]	2 [1–5]	0.28
ECMO support after surgery, n (%)	64 (21)	9 (22)	21 (16)	34 (26)	0.17
Duration of ECMO support, days, med [IQR]	0 [0–2]	0 [0–1]	0 [0–2]	0 [0–2]	0.15
Atrial fibrilation, n (%)	114 (38)	12 (29)	46 (36)	56 (43)	0.25
MOF syndrome, n (%)	100 (33)	14 (34)	39 (30)	47 (36)	0.59
Respiratory complications					
Duration of MV, med [IQR]	3 [1–17]	3[1–12]	3 [1–10]	4 [1–26]	0.091
PGD, n (%)	161 (53)	17 (41)	60 (46)	84 (63)	0.006
Grade 3 PGD	117 (38)	14 (34)	40 (31)	63 (47)	0.018
NBA during hospitalization in ICU, n (%)	89 (29)	8 (20)	30 (23)	51 (39)	0.008
Duration of NBA administration, days, med [IQR]	0 [0–1]	0 [0–0]	0 [0–1]	0 [0–2]	0.020
Prone positionning, n (%)	43 (14)	2 (5)	13 (10)	28 (21)	0.007
Number of sessions of prone positionning, med [IQR]	0 [0–0]	0 [0–0]	0 [0–0]	0 [0–0]	0.011
Extubation failure, n (%)	55 (22)	9 (27)	27 (25)	19 (18)	0.43
Tracheostomy for ventilation weaning, n (%)	85 (28)	9 (22)	27 (21)	49 (37)	0.008
Infectious complications					
Septic shock, n (%)	90 (30)	9 (22)	35 (27)	46 (35)	0.19
Number of pneumonias, med [IQR]	1 [1, 2]	1 [1, 2]	1 [1, 2]	1 [1, 2]	0.41
Renal complications					
AKI, n (%)	145 (48)	21 (51)	59 (46)	65 (49)	0.82
KDIGO score, med [IQR], n (%)	1 [0–2]	1 [0–2]	1 [0–2]	1 [0–3]	0.29
KDIGO 3, n (%)	60 (20)	9 (22)	17 (13)	34 (26)	0.036
Renal replacement therapy, n (%)	48 (16)	7 (17)	14 (11)	27 (20)	0.11
Surgical complications					
Thoracic surgical reintervention, n (%)	61 (20)	7 (17)	25 (19)	29 (22)	0.77
Abdominal surgery, n (%)	31 (10)	6 (15)	8 (6)	17 (13)	0.10
Other complications					
Bonchial anastomotic dehiscence, n (%)	40 (16)	2 (6)	14 (13)	24 (21)	0.072
Antibody-mediated rejection, n (%)	81 (27)	10 (24)	32 (25)	39 (30)	0.62
Acute cellular rejection, n (%)	40 (13)	5 (12)	13 (10)	22 (17)	0.28
Outcome					
Duration of ICU stay, days, med [IQR]	17 [9–31]	18 [10–28]	15 [10–26]	18 [11–41]	0.15
Death on day-90, n (%)	52 (17)	5 (12)	24 (18)	23 (17)	0.65
Death at one year, n (%)	86 (28)	9 (22)	37 (28)	40 (30)	0.60

Quantitative variables were compared using Mann-Withney U test; qualitative datas using Chi-2 tests

*SAPS II* simplified acute physiology score II, *SOFA* sequential organ failure assessment, *ICU* intensive care unit, *ECMO* extracorporeal membrane oxygenation, *MOF* multiorgan failure, *PGD* primary graft dysfunction, *NBA* neuroblocking agent, *AKI* acute kidney injury, *RRT* renal replacement therapy, *KDIGO* kidney disease improving global outcome

**Table 5:** Postoperative complications during hospitalization in the ICU and short-term outcomes of the recipients; donor BMI univariate analysis

	Overall, *N* = 279 (100%)	Donor underweight, *N* = 10 (3.6%)	Donor normal weight, *N* = 128 (46%)	Donor overweight, *N* = 95 (34%)	Donor obesity, *N* = 46 (16%)	*p* value
At admission in the ICU						
SAPS II at admission in ICU, med [IQR]	44 [39–53]	40 [36–48]	45 [39–53]	44 [39–53]	45 [41–52]	0.41
SOFA score at admission in ICU, med [IQR]	8 [7–10]	6 [6–9.3]	8 [7–10]	8 [7–10]	7.5 [6–10]	0.30
Serum lactate > 3 mmol/L	101 (36)	3 (30)	46 (36)	35 (37)	17 (37)	0.99
Serum lactate > 2 mmol/L	170 (61)	8 (80)	77 (60)	62 (65)	23 (50)	0.22
Hemodynamic status during hospitalization in ICU						
Duration of catecholamine support, med [IQR]	2 [1–4]	2 [1–7]	2 [1–4]	2 [1–4]	2 [1–3]	0.75
Atrial fibrillation, n (%)	103 (37)	7 (70)	39 (31)	43 (45)	14 (32)	0.020
MOF syndrome, n (%)	94 (34)	4 (40)	42 (33)	36 (38)	12 (26)	0.54
Duration of ECMO support	0 [0–2]	0 [0–0]	0 [0–2]	1 [0–3]	0 [0–0]	0.004
Respiratory complications						
Duration of MV, med [IQR]	3 [1–19]	5 [1–24]	3 [1–17]	3 [1–18]	3 [1–20]	0.98
PGD, n (%)	147 (53)	4 (40)	66 (52)	55 (58)	22 (48)	0.54
Grade 3 PGD, n (%)	74 (27)	2 (20)	36 (28)	30 (32)	6 (13)	0.10
NBA administration, n (%)	85 (31)	4 (40)	36 (29)	32 (34)	13 (28)	0.73
Duration of NBA administrationn, med [IQR]	3 [1–5]	5 [4–8]	3 [1–4]	3 [1–5]	2 [1–5]	0.45
Prone positionning, n (%)	41 (15)	3 (30)	17 (13)	16 (17)	5 (11)	0.37
Reintubation	50 (22)	2 (22)	26 (25)	11 (15)	11 (28)	0.28
Tracheostomy for ventilation weaning, n (%)	80 (29)	3 (30)	38 (30)	27 (28)	12 (26)	0.97
Infectious complications						
Bacteriemia, n (%)	44 (16)	3 (30)	18 (14)	17 (18)	6 (13)	0.50
Mediastinitis, n (%)	15 (5.4)	1 (10)	6 (4.7)	5 (5.3)	3 (6.5)	0.67
Septic shock	83 (30)	2 (20)	36 (28)	32 (34)	13 (29)	0.76
Number of pneumonia, med [IQR]	1 [1, 2]	1 [1–1.75]	1 [1, 2]	1 [1, 2]	1 [1, 2]	0.86
Surgical complications						
Surgical thoracic reintervention, n (%)	54 (19)	1 (10)	23 (18)	22 (23)	8 (17)	0.71
Abdominal surgery, n (%)	29 (10)	2 (20)	13 (10)	11 (12)	3 (6.5)	0.50
Renal complications						
AKI, n (%)	133 (48)	4 (40)	62 (49)	43 (45)	24 (52)	0.84
KDIGO stage, med [IQR]	1 [0–2]	0 [0–1]	1 [0–2]	1 [0–2]	1 [0–2]	0.50
Renal replacement therapy, n(%)	44 (16)	0 (0)	19 (15)	19 (20)	6 (13)	0.39
Other complications						
Bronchial anastomotic dehiscence, n(%)	38 (16)	2 (25)	17 (15)	9 (11)	10 (24)	0.17
Antibody mediated rejection, n (%)	75 (27)	2 (20)	36 (28)	27 (29)	10 (22)	0.83
Acute cellular rejection, n (%)	33 (12)	1 (10)	17 (13)	10 (11)	5 (11)	0.94
Outcome						
Duration of ICU stay, med IQR]	17 [9–32]	20 [9, 11–32]	17 [10–35]	18 [12–28]	15 [9–31]	0.77
Death on day-90, n (%)	49 (18)	0 (0)	21 (16)	20 (21)	8 (17)	0.45

Quantitative variables were compared using Mann-Withney U test; qualitative datas using Chi-2 tests

*SAPS II* simplified acute physiology score II, *SOFA* sequential organ failure assessment, *ICU* intensive care unit, *ECMO* extracorporeal membrane oxygenation, *MOF* multiorgan failure, *PGD* primary graft dysfunction, *NBA* neuroblocking agent, *AKI* acute kidney injury, *RRT* renal replacement therapy, *KDIGO*, kidney disease improving global outcome

The occurrence of PGD and grade 3 PGD were significantly more frequent in overweighted/obese recipients (p = 0.006 and p = 0.018 respectively). As a consequence, NBA administration and its duration (p = 0.008 and 0.020 respectively), and prone positioning (*p* = 0.007) were significantly associated to overweight/obesity. KDIGO 3 AKI was significantly more frequent in overweighted/obese recipients (p = 0.036). No significant difference was observed between the three groups concerning severity scores at ICU admission, haemodynamic status, nor infectious, surgical, airway complications and acute rejection.

Although grafts from overweight/obese donors had significantly lower PaO2/FiO2 ratios before organ donation than grafts from other donors, no difference was observed in early postoperative complications or in the recipients mortality rate.

### Short-term outcome according to the pretransplantation BMI of the recipients

**Fig.3:**
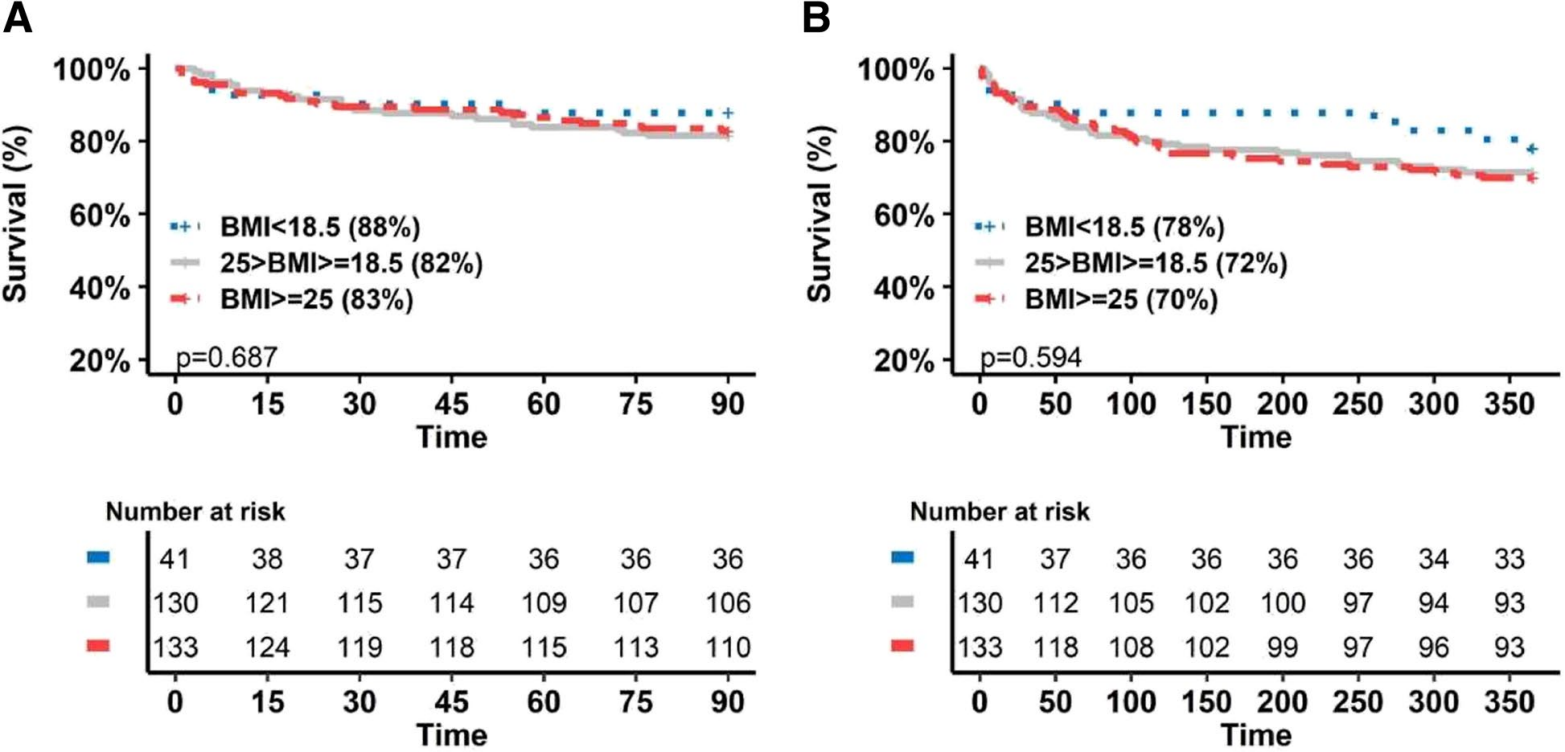
Short-term survival of LT recipients according to preoperative BMI. **A** 90-day survival **B** One-year survival

**Fig.4:**
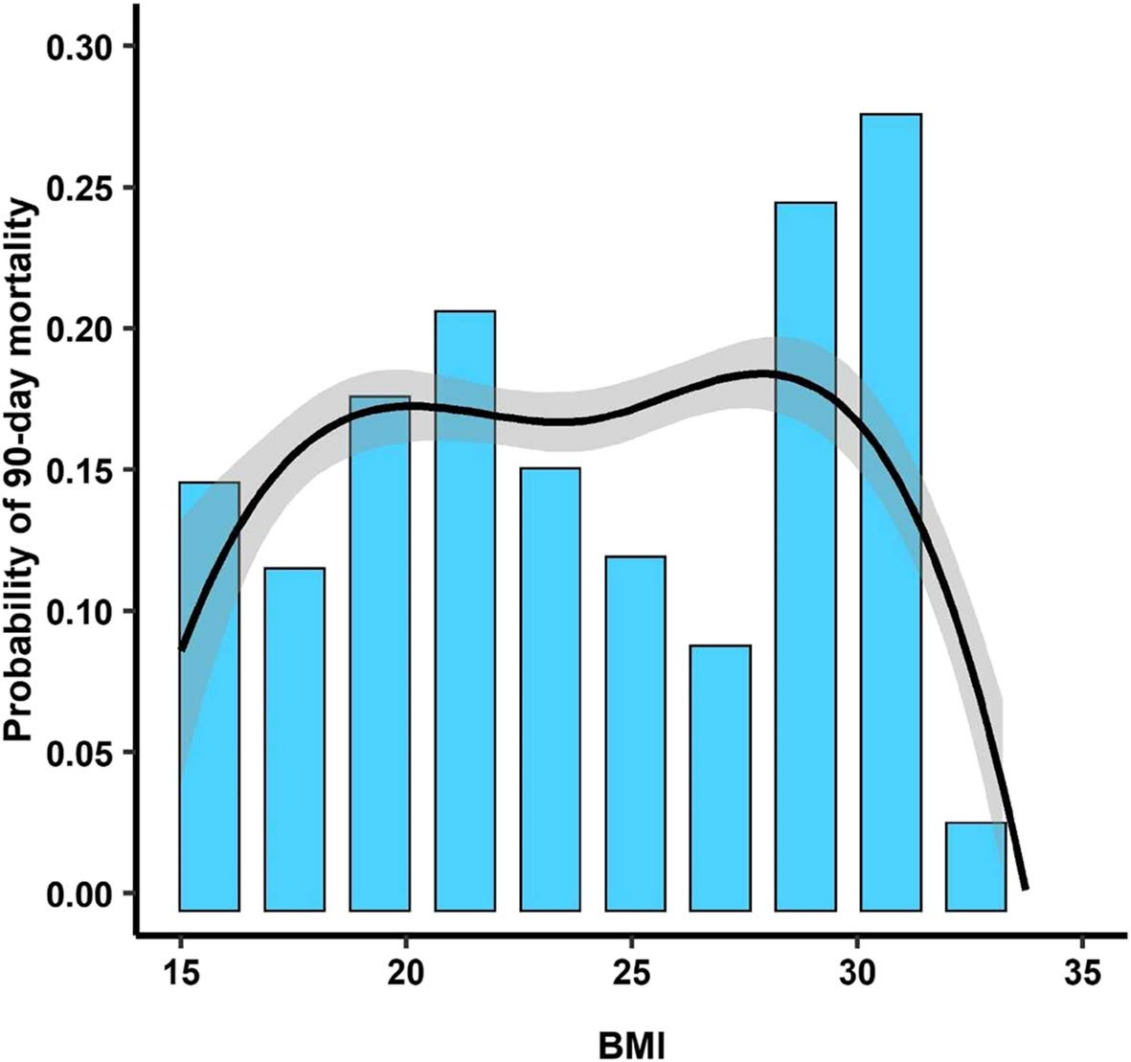
Probability of 90-day mortality depending on the recipient’s pretransplantation BMI

The independent risks factors for 90-day mortality in multivariate analysis are presented in Table 6. Pretransplantation overweight/obesity of the recipient was not an independent risk factor for 90-day mortality (*p* = 0.33). The short-term outcome of LT recipients depending of their pretransplantation BMI is presented in Fig. 3. The probability of 90-day mortality depending of the pretransplantation BMI is presented in Fig. 4.

**Table 6:** Risk factors for death on Day 90 according to multivariate analysis

	**OR**	**95%CI**	***p***
Tobbacco use (donor)	0.53	0.23 – 1.22	0.13
PaO2/FiO2 (donor)	0.97	0.92 – 1.01	0.13
Preoperative BMI ≥ 25 kg/m^2^	0.66	0.28 – 1.51	0.33
ECMO support during surgery	1.42	0.48 – 4.88	0.55
Dobutamine administration during surgery	7.52	0.75 – 137.47	0.12
Vascular filling ≥ 2500 mL	1.41	0.38 – 7.14	0.64
SAPS II score at ICU admission	1.16	1.11 – 1.21	< 0.001

Variables with a *p* value < 0.2 in univariate analysis were entered into a backward stepwise logistic regression mode

*OR* odds ratio*, 95% CI* 95% confidence interval*, BMI* body mass index*, ECMO* extracorporeal membrane oxygenation*, SAPS II* simplified acute physiology score*, ICU* intensive care unit

### Analysis of the pulmonary fibrosis / emphysema subpopulations

The characteristics of the pulmonary fibrosis and COPD subgroups and their postoperative complications during ICU stay are presented as supplemental data (Table S1, S2, S3, S4).

## Discussion

The main objective of this study was to assess the impact of abnormal pretransplantation BMI of recipients and donors on recipient’s outcome after LT, focusing on the early postoperative complications during hospitalization in ICU. In this monocentric cohort of 304 LT recipients, the need for ECMO support during surgery (*p* = 0.021) and early postoperative respiratory and renal complications (PGD, NBA administration and duration, prone positioning, need for tracheostomy for ventilation weaning, KDIGO 3 AKI) were significantly increased in overweighted or obese recipients. No difference appeared in haemodynamic status, infectious complications, duration of MV or of ICU stay, or 90-days mortality rate (*p* = 0.65). The grafts from overweighted or obese donors had significantly lower PaO2/FiO2 ratios before organ donation, but no difference was observed in the early postoperative complications or in mortality rate of recipients after LT.

A majority of LT recipients (57%) in our cohort presented an abnormal preoperative BMI, overweight being the most common disorder (32% of the all patients). This result is consistent with prior studies; Singer et al.study described BMI ≥ 25 kg/m^2^ in 50% of the cases [9]. In our cohort, recipient overweight/obesity was more frequent in recipients with pulmonary fibrosis, in agreement with prior literature [21].

The median BMI in our cohort was 24 [20–27] kg/m^2^, lower than the median BMI described by the last ISHLT report (26.5[19.6–34.6] Kg/m^2^) [22], whereas cystic fibrosis as indication for LT is poorly represented in our cohort (4(1%) patients), and these patients are frequently underweighted [21]. These findings probably reflect the strict adherence of the recipients to the ISHLT guidelines (class II and III obesity as an absolute contraindication) [12].

Our study showed that increased postoperative morbidity in overweight patients was exclusively linked to respiratory and renal complications. Indeed, no significant difference was observed in hemodynamic status or infectious, surgical or airway complications. Early postoperative complications associated with increased mortality in obese recipients have already been suspected. Two studies found no difference in survival when the analysis excluded recipients who died in the first year after LT, suggesting that overall mortality was linked to early complications [5, 8]. A recent study showed that recipients with a low BMI are at increased risk of death from infection, acute respiratory failure, and chronic lung allograft dysfunction, whereas recipients with a higher BMI are at increased risk of death from PGD, acute respiratory failure, and chronic lung allograft dysfunction [23].

The incidences of PGD and grade 3 PGD in our cohort were 53% and 38%, respectively. An increased risk of PGD in recipients with a preoperative BMI ≥ 25 kg/m2 has already been reported in ISHLT reports and described in some prior studies [24]. Lederer et al. showed that obesity is associated with increased risk of PGD occurrence [25], the severity of which is linked to decreased survival [26]. Chronic inflammatory status, linked to excess adipose tissue, could be responsible for the early postoperative plasmatic overexpression of proinflammatory cytokines and chemokines (MCP-1, IP-10) associated with PGD occurrence [27]. Several studies have described an association between high plasma leptine levels and PGD occurrence [9, 25], confirming the relationship between obesity and PGD.

In our study, pretransplantation overweight/obesity in the recipient was significantly associated with increased ECMO support during surgery. To our knowledge, this link has never been described in prior studies. This result is consistent with the increased occurrence of early respiratory complications after LT.

Despite the increase in respiratory complications in patients with preoperative overweight/obesity, 90-day and one-year mortality did not differ among the three groups. A prior retrospective study analyzed 5.978 patients and revealed that the mortality rate was 15% greater for underweight recipients, 15% greater for overweight recipients, and 22% greater for obese recipients than for patients with a normal pretransplantation BMI [3]. We can hypothesize that the relatively small size of our cohort can explain these results. Morevoer, a strict selection of recipients with abnormal BMI may also explain this result.

To our knowledge, no prior study has specifically assessed the impact of overweight and obesity in donors on early postoperative complications after LT. Our study showed that grafts from overweight or obese donors had a significantly lower PaO2/FiO2 ratio before organ donation, without any increase in morbidity or mortality after LT. A lower PaO2/FiO2 may be related to atelectasies under MV. Therefore, a lower PaO2/FiO2 ratio before organ donation may be tolerable in obese/overweighted donors without increasing the postoperative risk for the recipient.

In our cohort, the lowest probability of 90-day mortality was observed in patients whose pretransplantation BMI was between 26 and 27 kg/m^2^. This result must be considered carefully because of the relatively small size of our cohort. However, these findings are in accordance with those of the Fernandez et al. study, which assessed 17,000 patients between 2005 and 2016 and reported a significant reduction in 90-day and one-year mortality after LT in the subgroup of patients with pretransplantation BMIs of 25 kg/m2 and 26 kg/m2 [10] . Another monocentric study of 324 recipients revealed a significant decrease in the mortality rate in the overweight group compared with the normal weight group (*p* = 0.005), with a 50% reduction in mortality risk [11]. Singer et al. also showed that a lower probability of survival was observed in patients whose BMI was approximately 25 kg/m^2^ (*p* = 0.02) [9].

Interestingly, our study did not show any effect of pretransplantation underweight of the recipients on early postoperative complications or short-term mortality. This result contradicts the findings of other studies. Singer et al. observed that underweight was associated with a 35% increased relative risk of death at one year [9]. In this study, which assessed 9073 patients, 900 (10%) were underweight, and 439 (48.8%) were cystic fibrosis patients. This underlying disease was poorly represented in our cohort (4 (1%) recipients).

Our study has several limitations. First, the monocentric design, the relatively small size of the cohort, and the retrospective analysis of a prospective cohort limit the generalizability of the results. The small representation of cystic fibrosis in our cohort resulted in a small representation of underweight patients (6.6%) and limited the ability to detect differences in 90-day mortality and the impact of underweight on postoperative morbidity.

Second, the median BMI in the overweight/obese group was close to normal, and only 36 patients (12% of the cohort) had a BMI > 30 kg/m2. Similarly, the median pretransplant BMI in the underweight group was 17 [16–18] kg/m2, which was relatively close to normal. Only 11 (3.6%) patients had a BMI ≤ 16 kg/m2. We can hypothesize that this could explain why no effect was observed on mortality or duration of MV in patients with an abnormal BMI.

Third, BMI is an imperfect tool for determining underweight or overweight status. Indeed, it does not take into account body composition, sex (adipose tissue being more common in women than in men for the same BMI), age, or ethnicity. BMI cutoff values commonly used to diagnose obesity have high specificity but low sensitivity for identifying adiposity, as they fail to identify half of the people with excess adiposity [28]. In 2020, the Global Leadership Initiative on Malnutrition (GLIM) proposed integrating body composition data such as muscle loss or sarcopenia into the diagnostic process of underweight individuals [29]. Metabolic risk classifications such as the Adult Treatment Panel-III (ATP-III) [30] or the Karelis et al. criteria [31] are more sensitive for characterizing body composition or metabolic risk.

## Conclusion

In our monocentric retrospective study assessing early postoperative complications in LT recipients stratified by recipient and donor BMI, pretransplantation overweight or obesity was strongly associated with early respiratory complications and KDIGO 3 AKI occurrence during hospitalization in the ICU, without any difference in 90-day or one-year mortality. Donor’s overweight or obesity was associated with decreased PaO2/FiO2 ratio before organ donation, without any effect on postoperative complications or short-term mortality of the recipients.

### Supplementary Information

**Supplementary Material 1**.

## Data Availability

Data are available on request to the corresponding author.

## Abbreviations

AKI: Acute kidney injury
BMI: Body mass index
COPD: Chronic obstructive pulmonary disease
ECMO: Extracorporeal membrane oxygenation
ICU: Intensive care unit
ISHLT: International society for heart and lung transplantation
IPF: Idiopathic pulmonary fibrosis
KDIGO: Kidney disease: improving global outcome
LT: Lung transplantation
MOF: Multiorgan failure
MV: Mechanical ventilation
NBA: Neuroblocking agent
OR: Odds ratio
PGD: Primary graft dysfunction
PRC: Packed red cell
RRT: Renal replacement therapy
SOFA: Sepsis-related Organ Failure Assessment
