# Supplementary material for: Impact of recipient and donor pretransplantation body mass index on early postosperative complications after lung transplantation
Source: BMC Pulm Med. 2024 Apr 3;24:161. doi: 10.1186/s12890-024-02977-z (PMC10988822; doi:10.1186/s12890-024-02977-z)

**SUPPLEMENTAL DATA**

**TABLE S1:** Subgroup analysis of pulmonary fibrosis recipients according to pretransplantation BMI: univariate analysis

*BMI: body mass index; IQR: interquartile range; MV: mechanichal ventilation; RBC: red blood cell; FFP: fresh frozen plasma*

|  | **All cohort**,  N = 136 | **Recipient underweight**,  N = 9 (6.6%) | **Recipient normal weight**,  N = 50 (37%) | **Recipient overweight/obesity**,  N = 77 (57%) | **p** |
| --- | --- | --- | --- | --- | --- |
| **Characteristics of the donors** |  |  |  |  |  |
| Duration of MV, days, med [IQR] | 2 [1-4] | 1 [1-3] | 2 [1-4] | 2 [2-4] | 0.49 |
| PaO2/FiO2, donor, med [IQR] | 394 [334-463] | 400 [314-422] | 400 [328-450] | 385 [345-468] | 0.78 |
| Age, donor, med [IQR] | 51 [38-64] | 49 [46-56] | 50 [29-66] | 52 [40-60] | 0.98 |
| Tobbacco use, donor, n (%) | 50 (38) | 4 (44) | 8 (17) | 38 (49) | < 0.001 |
| Transfusion, donor, n (%) | 40 (30) | 1 (11) | 19 (40) | 20 (26) | 0.15 |
| **Characteristics of the recipients** |  |  |  |  |  |
| Age, recipient, years, | 58 [51-63] | 47 [43-58] | 59 [51-64] | 58 [53-63] | 0.099 |
| Male gender, n (%) | 105 (77) | 5 (56) | 38 (76) | 62 (81) | 0.22 |
| Height, cm, med [IQR] | 172 [166-178] | 163 [158-174] | 170 [165-176] | 173 [170-178] | 0.056 |
| Weight, kg, med [IQR] | 76 [65-84] | 45 [43-58] | 65 [59-70] | 82 [78-90] | < 0.001 |
| BMI, kg/m², med [IQR] | 25 [23-28] | 18 [17-18] | 23 [21-24] | 28 [26-30] | < 0.001 |
| Smoking history, recipient, n (%) | 82 (60) | 3 (33) | 28 (56) | 51 (66) | 0.11 |
| Comorbidities |  |  |  |  |  |
| Diabetes mellitus, n (%) | 16 (12) | 0 (0) | 5 (10) | 11 (14) | 0.63 |
| High blood pressure, n (%) | 33 (24) | 0 (0) | 9 (18) | 24 (31) | 0.054 |
| Hypercholesterolemia, n (%) | 33 (24) | 2 (22) | 10 (20) | 21 (27) | 0.66 |
| Ischemic heart disease, n (%) | 19 (14) | 1 (11) | 2 (4) | 16 (21) | 0.016 |
| Peripheral arterial disease, n (%) | 5 (4) | 0 (0) | 2 (4) | 3 (4) | >0.999 |
| Preoperative assesment |  |  |  |  |  |
| Pulmonary hypertension, n (%) | 69 (51) | 3 (38) | 28 (56) | 38 (49) | 0.54 |
| Dilatation of the right ventricle, n (%) | 43 (32) | 4 (44) | 18 (36) | 21 (27) | 0.39 |
| Left ventricular ejection fraction, med [IQR] | 63 [58-68] | 60 [53-66] | 65 [59-69] | 61 [58-65] | 0.37 |
| Serum creatinin, med [IQR] | 73 [62-84] | 57 [54-62] | 70 [57-80] | 76 [66-88] | 0.006 |
| Clinical characteristics before surgery |  |  |  |  |  |
| High flow oxygenotherapy before surgery, n (%) | 42 (31) | 3 (33) | 17 (34) | 22 (29) | 0.76 |
| ECMO as bridge to LT, n (%) | 15 (11) | 2 (22) | 4 (8) | 9 (12) | 0.36 |
| High emergency LT, n (%) | 46 (34) | 5 (56) | 19 (38) | 22 (29) | 0.19 |
| **Characteristics of intraoperative period** |  |  |  |  |  |
| Bilateral LT, n (%) | 74 (54) | 6 (67) | 30 (60) | 38 (49) | 0.43 |
| Retransplantation, n (%) | 0 (0) | 0 (0) | 0 (0) | 0 (0) |  |
| Duration of surgical procedure, med [IQR] | 420 [330-480] | 400 [300-420] | 420 [360-480] | 400 [330-490] | 0.83 |
| Peridural anesthesia, n (%) | 82 (60) | 3 (33) | 32 (64) | 47 (61) | 0.25 |
| ECMO support during surgery, n (%) | 114 (84) | 9 (100) | 40 (80) | 65 (84) | 0.43 |
| Cathecholamine support during surgery, n (%) | 133 (98) | 9 (100) | 48 (96) | 76 (99) | 0.64 |
| Dobutamine, n (%) | 5 (4) | 1 (11) | 2 (4) | 2 (3) | 0.31 |
| Transfusion during surgery, n (%) |  |  |  |  |  |
| RBC transfusion | 86 (63) | 7 (78) | 35 (70) | 44 (57) | 0.25 |
| 0 RBC unit | 48 (36) | 2 (22) | 15 (30) | 31 (41) | 0.55 |
| 1-4 RBC units | 66 (49) | 5 (56) | 28 (56) | 33 (43) |  |
| ≥ 5 RBC units | 21 (16) | 2 (22) | 7 (14) | 12 (16) |  |
| FFP transfusion | 81 (60) | 6 (67) | 29 (58) | 46 (60) | 0.93 |
| Platelet transfusion | 24 (18) | 3 (33) | 11 (22) | 10 (13) | 0.15 |
| Vascular filling ≥ 2500 mL during surgery, n (%) | 110 (81) | 8 (89) | 40 (80) | 62 (82) | 0.94 |

*Quantitative variables were compared using Mann-Withney U test; qualitative datas using Chi-2 tests.*

**TABLE S2:** Postoperative complications during hospitalization in the ICU and outcomes of pulmonary fibrosis recipients according to preoperative BMI according to univariate analysis

*BMI: body mass index; IQR: interquartile range; SAPS II: simplified acute physiology score II; SOFA: sequential organ failure assessment; ECMO: extracorporeal membrane oxygenation; MOF: multiorgan failure; MV: mechanichal ventilation; PGD: primary graft dysfunction; NBA: neuroblocking agent administration; AKI: acute kidney injury; KDIGO: kidney disease-improving global outcome; ICU: intensive care unit*

|  | **Overall**,  N = 136 | **Recipient underweight**  N = 9 (7%) | **Recipient normal weight**  N = 50 (37%) | **Recipient overweight/obseity**,  N = 77 (57%) | **p value** |
| --- | --- | --- | --- | --- | --- |
| At admission in ICU |  |  |  |  |  |
| SAPS II score, med [IQR] | 44 [39-53] | 39 [34-50] | 44 [39-53] | 45 [40-54] | 0.52 |
| SOFA score, med [IQR] | 8 [6-10] | 7 [6-9] | 8 [6-10] | 8 [6-11] | 0.70 |
| Lactatemia > 3 mmol/L, n (%) | 50 (37) | 4 (44) | 17 (34) | 29 (38) | 0.78 |
| Lactatemia > 2 mmol/L, n (%) | 84 (62) | 8 (89) | 31 (62) | 45 (58) | 0.23 |
| Hemodynamic status during hospitalization in ICU |  |  |  |  |  |
| Duration of catecholamine administration, days, med [IQR] | 2 [1-4] | 2 [1-3] | 2 [1-4] | 2 [1-4] | 0.83 |
| Duration of ECMO support, days, med [IQR] | 0 [0-2] | 0 [0-0] | 0 [0-2] | 0 [0-2] | 0.37 |
| Atrial fibrilation, n (%) | 50 (38) | 2 (22) | 16 (33) | 32 (43) | 0.36 |
| MOF syndrome, n (%) | 46 (34) | 3 (33) | 19 (38) | 24 (32) | 0.78 |
| Respiratory complications |  |  |  |  |  |
| Duration of MV, med [IQR] | 2 [1-4] | 1.5 [1-2] | 1 [1-3] | 2 [1-6] | 0.053 |
| PGD, n (%) | 81 (60) | 4 (44) | 27 (54) | 50 (65) | 0.31 |
| Grade 3 PGD | 40 (29) | 2 (22) | 13 (26) | 25 (32) | 0.20 |
| NBA during hospitalization in ICU, n (%) | 43(32) | 2 (22) | 13 (26) | 28 (37) | 0.39 |
| Duration of NBA administration, days, med [IQR] | 0 [0-2] | 0 [0-2] | 0 [0-2] | 0 [0-2] | 0.74 |
| Prone positionning, n (%) | 20 (15) | 0 (0) | 5 (10) | 15 (19) | 0.22 |
| Extubation failure, n (%) | 22 (20) | 1 (14) | 13 (31) | 8 (13) | 0.090 |
| Tracheostomy for ventilation weaning, n (%) | 37 (27) | 2 (22) | 11 (22) | 24 (32) | 0.51 |
| Infectious complications |  |  |  |  |  |
| Septic shock, n (%) | 40 (29) | 1 (11) | 15 (30) | 24 (31) | 0.55 |
| Number of pneumonias, med [IQR] | 1 [1-2] | 1 [1-1] | 1 [1-2] | 1 [0-2] | 0.67 |
| Renal complications |  |  |  |  |  |
| AKI, n (%) | 62 (46) | 4 (44) | 22 (46) | 36 (47) | >0.999 |
| KDIGO score, med [IQR] , n (%) | 1 [0-2] | 1 [0-1] | 1 [0-2] | 1 [0-2] | 0.90 |
| Renal replacement therapy, n (%) | 19 (14) | 1 (11) | 6 (12) | 12 (16) | 0.85 |
| Surgical complications |  |  |  |  |  |
| Thoracic surgical reintervention, n (%) | 24 (18) | 2 (22) | 10 (20) | 12 (16) | 0.71 |
| Abdominal surgery, n (%) | 12 (9) | 0 (0) | 4 (8) | 8 (10) | 0.80 |
| Other complications |  |  |  |  |  |
| Bonchial anastomotic dehiscence, n (%) | 24 (21) | 1 (14) | 7 (16) | 16 (24) | 0.70 |
| Antibody-mediated rejection, n (%) | 43 (32) | 3 (33) | 17 (34) | 23 (30) | 0.88 |
| Acute cellular rejection, n (%) | 18 (13) | 0 (0) | 6 (12) | 12 (16) | 0.57 |
| Outcome |  |  |  |  |  |
| Duration of ICU stay, days, med [IQR] | 16 [10-32] | 13 [9-24] | 16 [11-27] | 16 [10-40] | 0.56 |
| Death on day-90, n (%) | 25 (18) | 1 (11) | 11 (22) | 13 (17) | 0.68 |
| Death at one-year, n (%) | 42 (31) | 2 (22) | 17 (34) | 23 (30) | 0.80 |

*Quantitative variables were compared using Mann-Withney U test; qualitative datas using Chi-2 tests.*

**TABLE S3:** Preoperative characteristics of COPD recipients according to pretransplantation BMI: univariate analysis

*BMI: body mass index; IQR: interquartile range; MV: mechanichal ventilation; NIV: noninvasive ventilation; RBC: red blood cell; FFP: fresh frozen plasma*

|  | **Overall**,  N = 92 | **Recipient underweight**  N = 11 (12%) | **Recipient normal weight**  N = 45 (49%) | **Recipient overweight/obesity**,  N = 36 (39%) | **p value** |
| --- | --- | --- | --- | --- | --- |
| **Characteristics of the donors** |  |  |  |  |  |
| Duration of MV, days, med [IQR] | 2 [1-3] | 2 [1.5-2] | 2 [1-3] | 2 [1-3] | 0.83 |
| PaO2/FiO2, donor, med [IQR] | 398 [326-468] | 430 [400-500] | 375 [323-467] | 411 [328-463] | 0.39 |
| Age, donor, med [IQR] | 52 [40-59] | 51 [38-54] | 51 [35-59] | 55 [42-60] | 0.40 |
| Tobbacco use, donor, n (%) | 41 (46) | 8 (73) | 17 (38) | 16 (47) | 0.11 |
| Transfusion, donor, n (%) | 20 (22) | 3 (27) | 8 (18) | 9 (26) | 0.60 |
| **Characteristics of the recipients** |  |  |  |  |  |
| Age, recipient, years, | 58 [53.8-62] | 62 [53-63.5] | 58.0 [55-61] | 58 [54.5-62.0] | 0.91 |
| Male gender, n(%) | 58 (63) | 4 (36) | 28(62) | 26 (72) | 0.10 |
| Height, cm, med [IQR] | 171 [163-176] | 158 [158-164] | 170 [163-178] | 172 [168-176] | 0.013 |
| Weight, kg, med [IQR] | 68 [58-79] | 45 [43-49] | 64 [57-70] | 78 [74-88] | < 0.001 |
| BMI, kg/m², med [IQR] | 24 [20-26] | 18 [17-18] | 22 [20-23] | 27 [26-28.8] | <0.001 |
| Smoking history, recipient, n (%) | 88 (96) | 10 (91) | 42 (93) | 36 (100) | 0.23 |
| Comorbidities |  |  |  |  |  |
| Diabetes mellitus, n (%) | 7 (8) | 0 (0) | 2 (4) | 5 (14) | 0.22 |
| High blood pressure, n (%) | 27 (29) | 4 (36) | 8 (18) | 15 (42) | 0.058 |
| Hypercholesterolemia, n (%) | 26 (28) | 3 (27) | 9 (20) | 14 (39) | 0.18 |
| Ischemic heart disease, n (%) | 7 (8) | 0 (0) | 2 (4) | 5 (14) | 0.22 |
| Peripheral arterial disease, n (%) | 7 (8) | 1 (10) | 1 (2) | 5 (14) | 0.11 |
| Preoperative assesment |  |  |  |  |  |
| Pulmonary hypertension, n (%) | 49 (54) | 7 (70) | 23 (51) | 19 (53) | 0.55 |
| Dilatation of the right ventricle, n (%) | 22 (24) | 2 (18) | 11 (25) | 9 (25) | >0.999 |
| Left ventricular ejection fraction, med [IQR] | 62 [59-68] | 62 [56-66] | 65 [60-70] | 62 [58-67] | 0.63 |
| Serum creatinin, med [IQR] | 65 [52-80] | 49 [44-56] | 69 [50-80] | 68 [59-84] | 0.002 |
| Clinical characteristics before surgery |  |  |  |  |  |
| NIV, n (%) | 46 (53) | 6 (55) | 21 (50) | 19 (58) | 0.87 |
| High flow oxygenotherapy before surgery, n (%) | 1 (1) | 1 (9) | 0 (0) | 0 (0) | 0.12 |
| ECMO as bridge to LT, n (%) | 1 (1) | 1 (9) | 0 (0) | 0 (0) | 0.12 |
| High emergency LT, n (%) | 1 (1) | 1(9) | 0 (0) | 0 (0) | 0.12 |
| **Characteristics of intraoperative period** |  |  |  |  |  |
| Bilateral LT, n (%) | 72 (78) | 6 (55) | 35 (78) | 31 (86) | 0.077 |
| Retransplantation, n (%) | 0 (0) | 0 (0) | 0 (0) | 0 (0) |  |
| Duration of surgical procedure, med [IQR] | 420 [362-450] | 420 [345-435] | 420 [372-450] | 420 [360-488] | 0.77 |
| Peridural anesthesia, n (%) | 67 (75) | 6 (55) | 32 (74) | 29 (83) | 0.16 |
| ECMO support during surgery, n (%) | 50 (54) | 5 (45) | 23 (51) | 22 (61) | 0.55 |
| Cathecholamine support during surgery, n (%) | 87 (95) | 11 (100) | 41 (91) | 35 (97) | 0.42 |
| Dobutamine, n (%) | 1 (1) | 0 (0) | 0 (0) | 1 (3) | 0.51 |
| Transfusion during surgery, n (%) |  |  |  |  |  |
| RBC transfusion | 56 (62) | 8 (73) | 26 (59) | 22 (61) | 0.79 |
| 0 RBC unit | 37 (40) | 3 (27) | 20 (44) | 14 (39) | 0.69 |
| 1-4 RBC units | 47 (51) | 6 (55) | 22 (49) | 19 (53) |  |
| ≥ 5 RBC units | 8 (9) | 2 (18) | 3 (7) | 3 (8) |  |
| FFP transfusion | 50 (54) | 8 (73) | 21 (47) | 21 (58) | 0.28 |
| Platelet transfusion | 14 (15) | 2 (18) | 6 (13) | 6 (17) | 0.78 |
| Vascular filling ≥ 2500 mL during surgery, n (%) | 82 (90) | 9 (82) | 41 (91) | 32 (91) | 0.70 |

*Quantitative variables were compared using Mann-Withney U test; qualitative datas using Chi-2 tests*

**TABLE S4:** Postoperative complications during hospitalization in the ICU and outcome of COPD recipients according to their pretransplantation BMI; univariate analysis

*BMI: body mass index; IQR: interquartile range; SAPS II: simplified acute physiology score II; SOFA: sequential organ failure assessment; ECMO: extracorporeal membrane oxygenation; MOF: multiorgan failure; MV: mechanichal ventilation; PGD: primary graft dysfunction; NBA: neuroblocking agent administration; AKI: acute kidney injury; KDIGO: kidney disease-improving global outcome; ICU: intensive care unit*

|  | **Overall**,  N = 92 | **Recipient underweight**  N = 11 (12%) | **Recipient normal weight**  N = 45 (49%) | **Recipient overweight/obesity**,  N = 36 (39%) | **p value** |
| --- | --- | --- | --- | --- | --- |
| At admission in ICU |  |  |  |  |  |
| SAPS II score, med [IQR] | 42 [38-49] | 45 [42-49] | 42 [37-47] | 42 [38-53] | 0.21 |
| SOFA score, med [IQR] | 8 [6-9] | 7 [7-9] | 7 [6-9] | 8 [7-9] | 0.19 |
| Lactatemia > 3 mmol/L, n (%) | 27 (29) | 3 (27) | 12 (27) | 12 (33) | 0.85 |
| Lactatemia > 2 mmol/L, n (%) | 50 (54) | 6 (55) | 21 (47) | 23 (64) | 0.30 |
| Hemodynamic status during hospitalization in ICU |  |  |  |  |  |
| Duration of catecholamine administration, days, med [IQR] | 1.5 [1-3] | 2 [1-3.5] | 1 [1-2] | 2 [1-3] | 0.092 |
| Duration of ECMO support, days, med [IQR] | 0 [0-0] | 0 [0-0] | 0 [0-1] | 0 [0-1] | 0.90 |
| Atrial fibrilation, n (%) | 29 (32) | 4 (36) | 12 (27) | 13 (36) | 0.66 |
| MOF syndrome, n (%) | 23 (25) | 4 (36) | 8 (9) | 15 (43) | < 0.001 |
| Respiratory complications |  |  |  |  |  |
| Duration of MV, med [IQR] | 2 [1-8] | 3 [2-6] | 2 [1-4] | 2 [1-20] | 0.36 |
| PGD, n (%) | 34 (37) | 2 (18) | 11 (24) | 21 (58) | 0.003 |
| Grade 3 PGD | 15 (16) | 1 (9) | 4 (9) | 10 (28) | 0.070 |
| NBA during hospitalization in ICU, n (%) | 23 (25) | 3 (27) | 7 (16) | 13 (36) | 0.11 |
| Duration of NBA administration, days, med [IQR] | 2 [1-2] | 1 [1-2] | 1 [1-2] | 2 [1-3] | 0.41 |
| Prone positionning, n (%) | 11 (12) | 0 (0) | 3 (7) | 8 (22) | 0.074 |
| Extubation failure, n (%) | 17 (22) | 4 (40) | 5 (13) | 8 (28) | 0.11 |
| Tracheostomy for ventilation weaning, n (%) | 21 (23) | 3 (27) | 6 (13) | 12 (33) | 0.091 |
| Infectious complications |  |  |  |  |  |
| Septic shock, n (%) | 23 (25) | 4 (36) | 7 (16) | 12 (33) | 0.12 |
| Number of pneumonias, med [IQR] | 1 [1-2] | 1 [1-2] | 1 [1-1] | 1 [1-2] | 0.12 |
| Renal complications |  |  |  |  |  |
| AKI, n (%) | 42 (46) | 6 (55) | 18 (40) | 18 (50) | 0.55 |
| KDIGO score, med [IQR], n (%) | 1 [0-2] | 1 [0-2.5] | 1 [0-1] | 1 [0-3] | 0.15 |
| Renal replacement therapy, n (%) | 11 (12) | 2 (18) | 1 (2) | 8 (22) | 0.011 |
| Surgical complications |  |  |  |  |  |
| Thoracic surgical reintervention, n (%) | 12 (13) | 1 (9) | 2 (4) | 9 (25) | 0.016 |
| Abdominal surgery, n (%) | 9 (10) | 3 (27) | 3 (7) | 3 (8) | 0.13 |
| Other complications |  |  |  |  |  |
| Bonchial anastomotic dehiscence, n (%) | 10 (12) | 1 (11) | 2 (5) | 7 (22) | 0.081 |
| Antibody-mediated rejection, n (%) | 20 (22) | 2 (18) | 9 (20) | 9 (26) | 0.83 |
| Acute cellular rejection, n (%) | 10 (11) | 2 (18) | 4 (9) | 4 (11) | 0.65 |
| Outcome |  |  |  |  |  |
| Duration of ICU stay, days, med [IQR] | 16 [11-26] | 23 [10-34] | 14 [10-21] | 16 [11-38] | 0.48 |
| Death on day-90, n (%) | 11 (12) | 1 (9) | 3 (7) | 7 (19) | 0.17 |
| Death at one-year, n (%) | 20 (22) | 2 (18) | 8 (18) | 10 (28) | 0.52 |

*Quantitative variables were compared using Mann-Withney U test; qualitative datas using Chi-2 tests.*

**FIGURE S1** Short-term survival of COPD patients according to their pretransplantation BMI (A: 90-day mortality; B: One-year mortality)

**
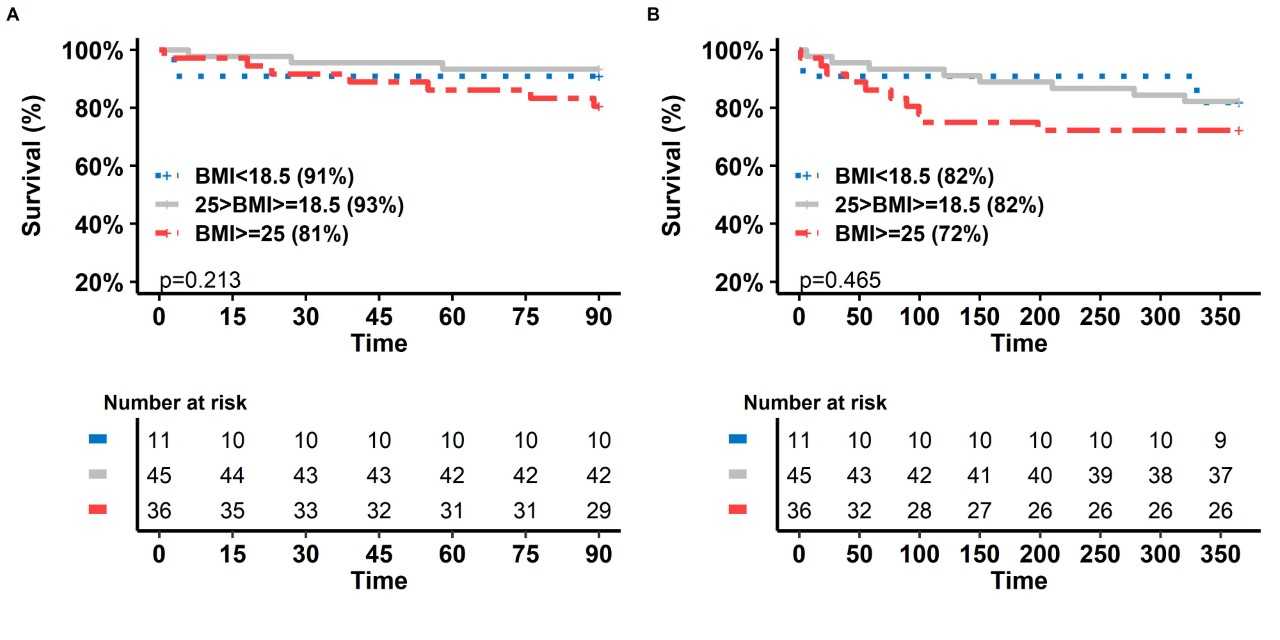
**

**FIGURE S2** Short-term survival of pulmonary fibrosis patients according to their pretransplantation BMI (A: 90-day mortality; B: One-year mortality)


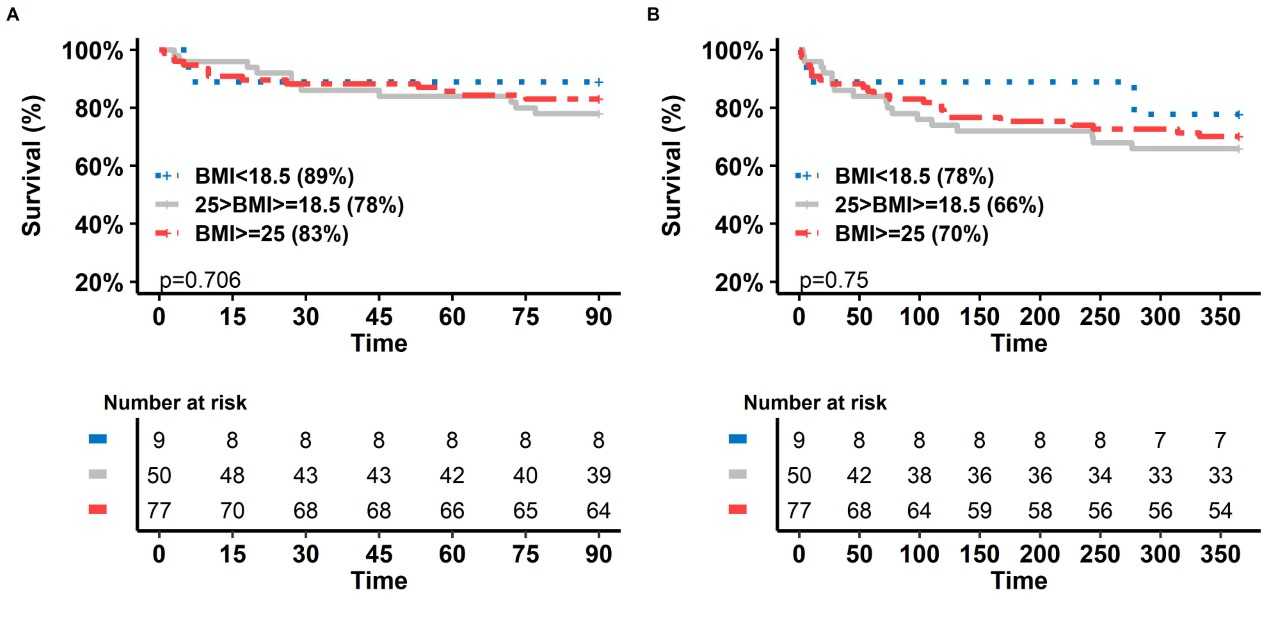

Supplement: Supplementary file 1 — Supplementary Material 1. [file 12890_2024_2977_MOESM1_ESM.docx]
